# Monogenic lupus – from gene to targeted therapy

**DOI:** 10.1186/s40348-024-00181-x

**Published:** 2024-09-12

**Authors:** Katharina Menzel, Kateryna Novotna, Nivya Jeyakumar, Christine Wolf, Min Ae Lee-Kirsch

**Affiliations:** 1 Department of Pediatrics, Medizinische Fakultät Carl Gustav Carus, Technische Universität Dresden, Dresden, 01307 Germany; 2 University Center for Rare Diseases, Medizinische Fakultät Carl Gustav Carus, Technische Universität Dresden, Dresden, 01307 Germany; 3 German Center for Child and Adolescent Health (DZKJ), Partner Site Leipzig/Dresden, Dresden, Germany

**Keywords:** SLE, Systemic lupus erythematosus, Cutaneous lupus erythematosus, Nucleic acid immunity, Nucleic acid metabolism, Nucleic acid sensing, Autoimmunity, Genetics, Pathogenesis, Targeted therapy, Type I interferon

## Abstract

Systemic lupus erythematosus (SLE) is a prototypic autoimmune disease characterized by loss of tolerance to nuclear antigens. The formation of autoantibodies and the deposition of immune complexes trigger inflammatory tissue damage that can affect any part of the body. In most cases, SLE is a complex disease involving multiple genetic and environmental factors. Despite advances in the treatment of SLE, there is currently no cure for SLE and patients are treated with immunosuppressive drugs with significant side effects. The elucidation of rare monogenic forms of SLE has provided invaluable insights into the molecular mechanisms underlying systemic autoimmunity. Harnessing this knowledge will facilitate the development of more refined and reliable biomarker profiles for diagnosis, therapeutic monitoring, and outcome prediction, and guide the development of novel targeted therapies not only for monogenic lupus, but also for complex SLE.

## Introduction

**Fig. 1:**
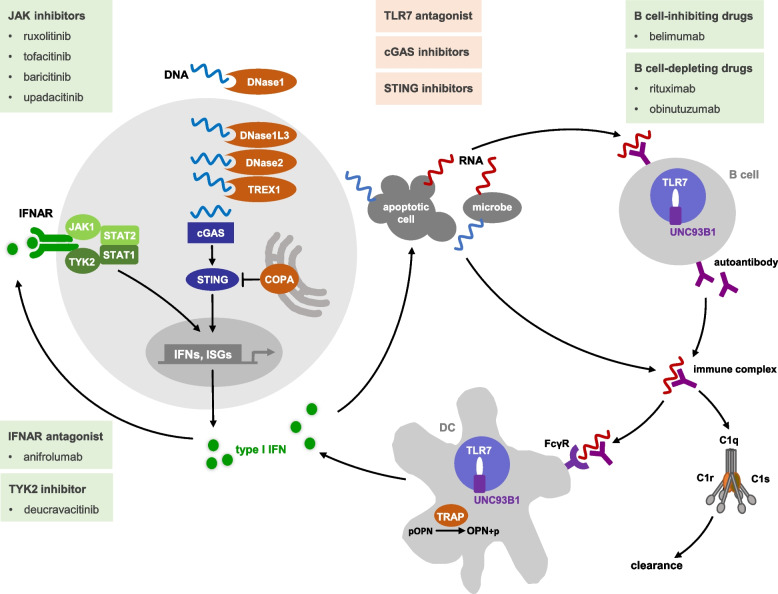
Pathogenetic principles of monogenic forms of lupus and therapeutic targets. Nucleases clear the extracellular space (DNase I) or the cytosol (DNase1L3, DNase2, TREX1) of DNA derived from pathogens or damaged cells or emanating from various metabolic processes to prevent aberrant immune recognition of self-DNA by the DNA sensor cGAS. Ligand binding of cGAS triggers activation of the STING signaling adaptor, resulting in transcriptional induction of type I IFN and IFN-stimulated genes (ISGs). Secreted type I IFN binds to the IFN receptor (IFNAR) which induces JAK/STAT signaling resulting in activation of IFN and ISG transcription. STING trafficking via COPI vesicles to the endoplasmic reticulum or into the autophagy pathway is required for termination of STING signaling. Gain-of-function mutations in STING or loss-of-function mutations in COPA result in ligand-independent cGAS activation with uncontrolled type I IFN activation. Gain-of-function mutations in the single-stranded RNA sensor TLR7 or its chaperone UNC93B1 result in constitutive type I IFN signaling and promote the proliferation of autoreactive B cells. Secreted autoantibodies form immune complexes that are cleared by complement activation. Loss-of-function mutations in the subunits encoding complement component 1 impair clearance of RNA autoantigen-containing immune complexes, which subsequently stimulate increased type I IFN production by dendritic cells (DC) after Fc gamma receptor (FcγR)-mediated internalization in a TLR7-dependent manner. TRAP dephosphorylates and thereby inactivates osteopontin (OPN), which promotes type I IFN production in plasmacytoid dendritic cells. Loss-of-function mutations in TRAP result in constitutively active OPN. Drugs that target specific molecules or pathways are shown in boxes. Green boxes indicate approved drugs, orange boxes indicate drugs in preclinical development or clinical trials

Systemic lupus erythematosus (SLE) is a complex autoimmune disease with a multifactorial etiology in which the interaction of multiple genes with environmental factors determines disease susceptibility [1]. The clinical spectrum of SLE is very broad, ranging from mild disease, which may be limited to skin and joint involvement, to life-threatening manifestations with renal impairment, severe cytopenias, central nervous system disease, and thromboembolic events. However, these clinically heterogeneous diseases converge on a common phenotype characterized by chronic overproduction of type I interferon (IFN), indicating that inappropriate activation of antiviral immunity is key to SLE pathogenesis [2]. Type I IFNs (IFN-α, IFN-β), which are induced by the activation of nucleic acid-sensing receptors of the innate immune system, act in an autocrine and paracrine manner by binding to the interferon-α receptor (IFNAR), a cell surface receptor composed of two subunits, IFNAR1 and IFNAR2 (Fig. 1) [3]. Type I IFN signaling activates the Janus kinase (JAK)—signal transducer and activator of transcription (STAT) pathway, leading to transcription of the IFN genes and of IFN-stimulated genes (ISGs). As a result of the ensuing transcriptional program, type I IFNs exert potent immunostimulatory effects promoting inflammation, the loss of B cell self-tolerance, and the formation of autoantibodies, often directed against nuclear self-antigens, including nucleic acids [2]. These antinuclear antibodies form complexes with antigens released from dying cells. Deposition of immune complexes in the capillary bed, followed by local complement and leukocyte activation, leads to destructive tissue inflammation [4]. In addition, these immune complexes provide an important stimulus for increased type I IFN production by dendritic cells, further fueling the autoimmune response (Fig. 1) [2].

Genome-wide association studies have provided important insights into the genetic architecture of SLE and identified numerous risk loci [5]. However, the causative disease genes at many loci remain unclear, limiting the translation of such genetic discoveries into novel therapeutic concepts. Recent advances in human genetics have led to the identification of rare monogenic causes of lupus, providing a unique opportunity to study the functional consequences of single gene mutations and to distinguish primary pathophysiological from secondary adaptive processes in SLE. In addition, unlike common complex SLE, which primarily affects women of childbearing age, monogenic lupus typically affects children of both sexes equally and is associated with a more severe disease phenotype, reflecting the strong genetic contribution. In particular, mutations in genes involved in nucleic acid metabolism, nucleic acid sensing, and type I IFN signaling have emerged as important causes of monogenic lupus, highlighting the central role of innate immunity in disease pathogenesis. Thus, by studying these rare Mendelian forms of SLE, it is possible to trace the pleotropic effects of a single gene and apply mechanistic insights to common forms of SLE. This review focuses on monogenic forms of lupus sensu stricto. Other inborn errors of immunity, in which immune dysregulation may be associated with lupus-like features, are not the subject of this review.

### Complement activation

Activation of complement component C1, consisting of C1q, C1r, and C1s, initiates the classical complement pathway. This occurs by binding of C1q to immune complexes, apoptotic bodies, or pathogens, leading to autocatalytic activation of C1r and subsequent cleavage of the zymogen C1s [6]. The classical complement pathway is involved in pathogen recognition, antibody-mediated cytotoxicity, and clearance of immune complexes and apoptotic debris (Fig. 1) [6]. The clearance of extracellular waste by the complement system is an important aspect of immune homeostasis, as reduced complement activity promotes presentation of autoantigens to the immune system in the context of inflammatory injury.

Deficiencies in the subcomponents of complement 1 (Table 1) result in early-onset SLE with prominent cutaneous disease, renal, and neurological symptoms [7, 8]. This is in contrast to deficiencies in other Mendelian forms of complement deficiency, in which systemic autoimmunity is associated with increased susceptibility to bacterial infection. In patients with complete C1 deficiency, autoimmunity is induced by increased neutrophil extracellular trap formation and enhanced activity of endosomal Toll-like receptor (TLR) 7 and TLR9, which sense RNA and DNA, respectively, upon Fc gamma receptor (FcγR)-mediated uptake of nucleic acid-containing immune complexes (Fig. 1) [7, 8]. This results in a self-perpetuating feedback loop that stimulates type I IFN activation and proliferation of autoreactive B cells.

**Table 1:** Monogenic forms of lupus. The table shows Mendelian diseases in which systemic or cutaneous lupus is the main clinical feature, with the exception of COPA, which is associated with interstitial lung disease, and SPENCD, which is associated with skeletal dysplasia. AR: autosomal recessive; AD: autosomal dominant; XD: X-linked dominant

Disease	Main clinical findings	Age of onset	Gene / Protein	Mode of inheritance	References
C1 complement deficiency	SLE	< 5 years	*C1R* (complement component C1r) *C1QA* (complement component C1q, A chain) *C1QB* (complement component C1q, B chain) *C1QC* (complement component C1q, C chain) *C1S* (complement component C1s)	AR	[7, 8]
DNase I deficiency	SLE	< 10 years, variable	*DNASE1* (deoxyribonuclease I)	AD	[10]
DNase I-like 3 deficiency	SLE	< 10 years, variable	*DNASE1L3* (deoxyribonuclease I-like 3)	AR	[13]
Autoinflammatory-pancytopenia syndrome (AIPCS)	anemia, thrombocytopenia, arthritis, dermatitis, hepatosplenomegaly, glomerulonephritis, fever	< 5 years	*DNASE2* (deoxyribonuclease II, lysosomal)	AR	[14]
Familial chilblain lupus (CHBL)	Chilblain lesions, arthralgia	< 5 years	*TREX1* (three prime repair exonuclease 1) *STING* (stimulator of interferon genes protein)	AD AD	[17, 18, 27]
TLR7 gain-of-function	SLE, autoimmune cytopenia, autoimmune encephalitis	< 5 years	*TLR7* (Toll-like receptor 7)	XD	[31, 32]
UNC93B1 gain-of-function	SLE, autoimmune cytopenias	< 5 years	*UNC93B1* (UNC93 homolog B1)	AD, AR	[33–37]
Autoimmune interstitial lung, joint, kidney disease (AILJK)	interstitial lung diseae, pulmonary hemorrhage, arthritis, nephritis	< 10 years	*COPA* (coatomer protein complex, subunit alpha)	AD	[28]
Spondyloenchondrodysplasia (SPENCD)	spondylometaphyseal dysplasia, arthritis, thrombocytopenia, basal ganglia calcification, spasticity	< 5 years, variable	*ACP5* (tartrate-resistant acid phosphatase, type 5)	AR	[38]

### Nucleic acid metabolism

Nucleolytic degradation of endogenous nucleic acids plays an important role in protecting against inappropriate and pathogenic activation of innate sensors by self-nucleic acids [9]. Heterozygous loss-of-function mutations in *DNASE1*, which encodes the major serum DNA-degrading enzyme, cause SLE associated with high levels of autoantibodies to nucleosomal antigens (Table 1) [10]. The DNA substrate for DNase I can come from a variety of sources, including extracellular DNA waste derived from dying cells or immune complexes and oxidized mitochondrial DNA contained in neutrophil extracellular traps [11]. While perturbations in the removal of extracellular nucleic acid waste primarily trigger type I IFN signaling through non-cell autonomous pathways, intracellular nucleic acids can also elicit antiviral immune responses in a cell-intrinsic manner. The host organism has therefore evolved several mechanisms to prevent harmful immune recognition of self-nucleic acids produced during physiological metabolic processes.

For example, biallelic loss-of-function mutations in *DNASE1L3*, which encodes an intracellular DNase involved in chromatin degradation during apoptosis, have been reported to cause familial SLE with renal involvement (Table 1) [12, 13]. Complete deficiency of DNase II, which is essential for endolysosomal degradation of DNA in macrophages, is associated with systemic and multi-organ autoimmunity (Table 1) [14]. Interestingly, type I IFN activation in DNase II deficiency is initiated by the DNA sensor, cyclic GMP-AMP synthase (cGAS), which recognizes undegraded lysosomal DNA released into the cytosol [15]. Mutations in the intracellular DNase TREX1 cause a spectrum of inflammatory phenotypes characterized by autoimmunity, including Aicardi-Goutières syndrome, a neuroinflammatory interferonopathy and familial chilblain lupus, a monogenic form of cutaneous lupus characterized by bluish-red, partially ulcerating, skin lesions at acral location (Table 1) [16–18]. Furthermore, rare *TREX1* variants contribute to the genetic risk of complex SLE [19, 20]. TREX1 is an outer nuclear membrane-anchored cytosolic DNase that degrades DNA metabolites resulting from DNA damage repair or from reverse transcribed retroelements to prevent cGAS-dependent innate immune activation (Fig. 1) [21–23]. Taken together, these findings have revealed novel cell-intrinsic mechanisms for the initiation of autoimmunity.

### Nucleic acid sensing and type I IFN signaling

cGAS acts as a central sensor of cytosolic DNA derived from pathogens or cellular stress. Upon DNA binding, cGAS catalyzes the synthesis of the second messenger, cyclic GMP-AMP (cGAMP), which binds and activates the adapter molecule stimulator of interferon genes (STING) localized at the endoplasmic reticulum membrane (Fig. 1) [24]. STING is then incorporated into coatomer protein complex II (COPII) vesicles and passes through the Golgi network, where it ultimately activates IFN regulatory factor 3 (IRF3), which induces gene expression of type I IFN, ISGs, and other proinflammatory cytokines. Termination of STING signaling occurs via autophagy-associated degradation of STING in the lysosome [24].

De novo gain-of-function mutations in *STING* underlie STING-associated vasculopathy (SAVI), an autoinflammatory interferonopathy characterized by acral vasculitis, recurrent fever, and interstitial lung disease due to constitutive type I IFN signaling even in the absence of cGAMP stimulation [25]. While patients with SAVI show no signs of autoimmunity, dominant *STING* mutations can also cause SLE-like disease and familial chilblain lupus (Table 1) [26, 27]. Heterozygous mutations in *COPA*, which encodes the alpha subunit of the COPI complex, cause COPA syndrome, an interferonopathy characterized by interstitial lung disease, arthritis, and immune complex-mediated glomerulonephritis [28]. The COPI complex regulates the sorting of molecules between Golgi cisternae and their transport from the Golgi to the endoplasmic reticulum. *COPA* mutations impair trafficking of STING-containing COPI vesicles to the endoplasmic reticulum and within the Golgi network, thereby promoting ligand-independent STING activation or preventing STING signaling termination [29, 30].

Unlike cGAS, which recognizes DNA in the cytosol, TLR7 senses endocytosed RNA in the endosomal compartment and triggers type I IFN activation via myeloid differentiation primary response gene 88 (MyD88) (Fig. 1). Gain-of-function mutations in *TLR7* have recently been shown to cause SLE due to constitutive type I IFN signaling (Table 1) [31, 32]. Similarly, mutations in *UNC93B1*, a chaperone that controls the trafficking and positioning of TLR7 within the endosomal membrane, have also been shown to cause early-onset SLE (Table 1) [33–37]. Indeed, these discoveries have shown that UNC93B1 controls ligand recognition, signaling initiation and signaling termination of the TLR7 pathway [33, 34]. Both, *TLR7* and *UNC93B1* mutations are associated with proliferation of autoreactive B cells, resulting in high levels of antinuclear antibodies.

SLE-like features are also seen in spondyloenchondrodysplasia (SPENCD), a skeletal dysplasia caused by biallelic mutations in *ACP5*, which encodes tartrate-resistant acid phosphatase (TRAP) [38]. In fact, patients may present with lupus while the bone changes may be subtle. TRAP dephosphorylates and inactivates osteopontin, a cytokine that has been shown to be essential for TLR9-dependent IFN-α production in plasmacytoid dendritic cells [39].

In summary, these findings have advanced our understanding of the multiple levels of both positive and negative regulatory control that govern type I IFN signaling during immune activation.

### Translational aspects – on the way to precision medicine for lupus

The genetic dissection of rare monogenic forms of lupus has provided unprecedented insight into the molecular basis of autoimmunity. Indeed, SLE is a heterogeneous group of diseases, not only clinically but also molecularly. As we continue to understand each pathway of the complex regulatory network that controls and fine-tunes the initiation, execution, and termination of immune responses, we will be able to apply this knowledge to the development of improved and more precise therapeutic concepts. The current arsenal of biologic disease-modifying antirheumatic drugs already provides a number of drugs such as JAK inhibitors, the IFNAR antagonist anifrolumab or B-cell targeting drugs that allow pathway-specific intervention (Fig. 1). Ongoing efforts to develop novel compounds targeting other molecules or pathways along the type I IFN signaling axis, such as cGAS/STING or TLR7, will undoubtedly broaden the prospects for targeted therapies. These efforts will be greatly aided by the definition of molecular profiles that will help predict who is at risk of complications and who is likely to respond to a given treatment. In addition, such molecular profiles will allow patient stratification for much-needed clinical trials based on mechanistic insights.

## Data Availability

Not applicable.

## Abbreviations

cGAS: Cyclic GMP-AMP synthase
COPA: Coatomer protein complex, subunit alpha
COPI/II: Coatomer protein complex I/II
cGAMP: Cyclic GMP-AMP
DNA/RNA: Deoxyribonucleic acid / ribonucleic acid
DNase: DNA-degrading nuclease
FcγR: Fc gamma receptor
IFN: Interferon
IFNAR: IFN-α/β receptor
IRF3: IFN regulatory factor 3
ISG: IFN-stimulated gene
JAK1: Janus kinase 1
MyD88: Myeloid differentiation primary response gene 88
OPN: Osteopontin
STAT1/2: Signal transducer and activator of transcription 1/2
STING: Stimulator of interferon genes
TLR7/9: Toll-like receptor 7/9
TRAP: Tartrate-resistant acid phosphatase
TYK2: Tyrosine kinase 2
UNC93B1: UNC93 homolog B1, TLR signaling regulator

## Data availability

No datasets were generated or analysed during the current study.

## References

[1] Kaul A, Gordon C, Crow MK, Touma Z, Urowitz MB, van Vollenhoven R, Ruiz-Irastorza G, Hughes G (2016) Systemic lupus erythematosus. Nat Rev Dis Primer 2:16039. 10.1038/nrdp.2016.39 10.1038/nrdp.2016.39 27306639

[2] Marshak-Rothstein A (2006) Toll-like receptors in systemic autoimmune disease. NatRevImmunol 6:823–835 10.1038/nri1957 PMC7097510 17063184

[3] Ivashkiv LB, Donlin LT (2014) Regulation of type I interferon responses. NatRevImmunol 14:36–49 10.1038/nri3581 PMC4084561 24362405

[4] Lovgren T, Eloranta ML, Bave U, Alm GV, Ronnblom L (2004) Induction of interferon-alpha production in plasmacytoid dendritic cells by immune complexes containing nucleic acid released by necrotic or late apoptotic cells and lupus IgG. Arthritis Rheum 50:1861–1872 15188363 10.1002/art.20254

[5] Guga S, Wang Y, Graham DC, Vyse TJ (2023) A review of genetic risk in systemic lupus erythematosus. Expert Rev Clin Immunol 19:1247–1258. 10.1080/1744666X.2023.2240959 37496418 10.1080/1744666X.2023.2240959

[6] Lintner KE, Wu YL, Yang Y, Spencer CH, Hauptmann G, Hebert LA, Atkinson JP, Yu CY (2016) Early components of the complement classical activation pathway in human systemic autoimmune diseases. Front Immunol 7:36. 10.3389/fimmu.2016.00036 26913032 10.3389/fimmu.2016.00036 PMC4753731

[7] Demirkaya E, Zhou Q, Smith CK, Ombrello MJ, Deuitch N, Tsai WL, Hoffmann P, Remmers EF, Takeuchi M, Park YH, Chae J, Barut K, Simsek D, Adrovic A, Sahin S, Caliskan S, Chandrasekharappa SC, Hasni SA, Ombrello AK, Gadina M, Kastner DL, Kaplan MJ, Kasapcopur O, Aksentijevich I (2017) Brief report: deficiency of complement 1r subcomponent in early-onset systemic lupus erythematosus: the role of disease-modifying alleles in a monogenic disease. Arthritis Rheumatol Hoboken NJ 69:1832–1839. 10.1002/art.40158 10.1002/art.40158 PMC5609811 28544690

[8] Wolf C, Brück N, Koss S, Griep C, Kirschfink M, Palm-Beden K, Fang M, Röber N, Winkler S, Berner R, Latz E, Günther C, Lee-Kirsch MA (2020) Janus kinase inhibition in complement component 1 deficiency. J Allergy Clin Immunol 146:1439–1442.e5. 10.1016/j.jaci.2020.04.002 32325142 10.1016/j.jaci.2020.04.002

[9] Schlee M, Hartmann G (2016) Discriminating self from non-self in nucleic acid sensing. Nat Rev Immunol 16:566–580. 10.1038/nri.2016.78 27455396 10.1038/nri.2016.78 PMC7097691

[10] Yasutomo K, Horiuchi T, Kagami S, Tsukamoto H, Hashimura C, Urushihara M, Kuroda Y (2001) Mutation of DNASE1 in people with systemic lupus erythematosus. NatGenet 28:313–314 10.1038/91070 11479590

[11] Caielli S, Athale S, Domic B, Murat E, Chandra M, Banchereau R, Baisch J, Phelps K, Clayton S, Gong M, Wright T, Punaro M, Palucka K, Guiducci C, Banchereau J, Pascual V (2016) Oxidized mitochondrial nucleoids released by neutrophils drive type I interferon production in human lupus. J Exp Med 213:697–713. 10.1084/jem.20151876 27091841 10.1084/jem.20151876 PMC4854735

[12] Errami Y, Naura AS, Kim H, Ju J, Suzuki Y, El-Bahrawy AH, Ghonim MA, Hemeida RA, Mansy MS, Zhang J, Xu M, Smulson ME, Brim H, Boulares AH (2013) Apoptotic DNA fragmentation may be a cooperative activity between caspase-activated deoxyribonuclease and the poly(ADP-ribose) polymerase-regulated DNAS1L3, an endoplasmic reticulum-localized endonuclease that translocates to the nucleus during apoptosis. J Biol Chem 288:3460–3468. 10.1074/jbc.M112.423061 23229555 10.1074/jbc.M112.423061 PMC3561564

[13] Al-Mayouf SM, Sunker A, Abdwani R, Abrawi SA, Almurshedi F, Alhashmi N, Al SA, Sewairi W, Qari A, Abdallah E, Al-Owain M, Al MS, Al-Rayes H, Hashem M, Khalak H, Al-Jebali L, Alkuraya FS (2011) Loss-of-function variant in DNASE1L3 causes a familial form of systemic lupus erythematosus. NatGenet 43:1186–1188 10.1038/ng.975 22019780

[14] Rodero MP, Tesser A, Bartok E, Rice GI, Della Mina E, Depp M, Beitz B, Bondet V, Cagnard N, Duffy D, Dussiot M, Frémond M-L, Gattorno M, Guillem F, Kitabayashi N, Porcheray F, Rieux-Laucat F, Seabra L, Uggenti C, Volpi S, Zeef LAH, Alyanakian M-A, Beltrand J, Bianco AM, Boddaert N, Brouzes C, Candon S, Caorsi R, Charbit M, Fabre M, Faletra F, Girard M, Harroche A, Hartmann E, Lasne D, Marcuzzi A, Neven B, Nitschke P, Pascreau T, Pastore S, Picard C, Picco P, Piscianz E, Polak M, Quartier P, Rabant M, Stocco G, Taddio A, Uettwiller F, Valencic E, Vozzi D, Hartmann G, Barchet W, Hermine O, Bader-Meunier B, Tommasini A, Crow YJ (2017) Type I interferon-mediated autoinflammation due to DNase II deficiency. Nat Commun 8:2176. 10.1038/s41467-017-01932-3 29259162 10.1038/s41467-017-01932-3 PMC5736616

[15] Gao D, Li T, Li XD, Chen X, Li QZ, Wight-Carter M, Chen ZJ (2015) Activation of cyclic GMP-AMP synthase by self-DNA causes autoimmune diseases. ProcNatlAcadSciUSA 112:E5699–E5705 10.1073/pnas.1516465112 PMC4620884 26371324

[16] Crow YJ, Hayward BE, Parmar R, Robins P, Leitch A, Ali M, Black DN, van BH, Brunner HG, Hamel BC, Corry PC, Cowan FM, Frints SG, Klepper J, Livingston JH, Lynch SA, Massey RF, Meritet JF, Michaud JL, Ponsot G, Voit T, Lebon P, Bonthron DT, Jackson AP, Barnes DE, Lindahl T (2006) Mutations in the gene encoding the 3’-5’ DNA exonuclease TREX1 cause Aicardi-Goutieres syndrome at the AGS1 locus. NatGenet 38:917–920 10.1038/ng1845 16845398

[17] Rice G, Newman WG, Dean J, Patrick T, Parmar R, Flintoff K, Robins P, Harvey S, Hollis T, O’hara A, Herrick AL, Bowden AP, Perrino FW, Lindahl T, Barnes DE, Crow YJ (2007) Heterozygous Mutations in TREX1 Cause Familial Chilblain Lupus and Dominant Aicardi-Goutieres Syndrome. AmJHumGenet 80:811–815 10.1086/513443 PMC1852703 17357087

[18] Lee-Kirsch MA, Chowdhury D, Harvey S, Gong M, Senenko L, Engel K, Pfeiffer C, Hollis T, Gahr M, Perrino FW, Lieberman J, Hubner N (2007) A mutation in TREX1 that impairs susceptibility to granzyme A-mediated cell death underlies familial chilblain lupus. JMolMed 85:531–537 10.1007/s00109-007-0199-9 17440703

[19] Lee-Kirsch MA, Gong M, Chowdhury D, Senenko L, Engel K, Lee YA, de SU, Bailey SL, Witte T, Vyse TJ, Kere J, Pfeiffer C, Harvey S, Wong A, Koskenmies S, Hummel O, Rohde K, Schmidt RE, Dominiczak AF, Gahr M, Hollis T, Perrino FW, Lieberman J, Hubner N (2007) Mutations in the gene encoding the 3’-5’ DNA exonuclease TREX1 are associated with systemic lupus erythematosus. NatGenet 39:1065–1067 10.1038/ng2091 17660818

[20] Namjou B, Kothari PH, Kelly JA, Glenn SB, Ojwang JO, Adler A, arcon-Riquelme ME, Gallant CJ, Boackle SA, Criswell LA, Kimberly RP, Brown E, Edberg J, Stevens AM, Jacob CO, Tsao BP, Gilkeson GS, Kamen DL, Merrill JT, Petri M, Goldman RR, Vila LM, Anaya JM, Niewold TB, Martin J, Pons-Estel BA, Sabio JM, Callejas JL, Vyse TJ, Bae SC, Perrino FW, Freedman BI, Scofield RH, Moser KL, Gaffney PM, James JA, Langefeld CD, Kaufman KM, Harley JB, Atkinson JP (2011) Evaluation of the TREX1 gene in a large multi-ancestral lupus cohort. Genes Immun 12:270–279 21270825 10.1038/gene.2010.73 PMC3107387

[21] Wolf C, Rapp A, Berndt N, Staroske W, Schuster M, Dobrick-Mattheuer M, Kretschmer S, König N, Kurth T, Wieczorek D, Kast K, Cardoso MC, Günther C, Lee-Kirsch MA (2016) RPA and Rad51 constitute a cell intrinsic mechanism to protect the cytosol from self DNA. Nat Commun 7:11752. 10.1038/ncomms11752 27230542 10.1038/ncomms11752 PMC4895045

[22] Stetson DB, Ko JS, Heidmann T, Medzhitov R (2008) Trex1 prevents cell-intrinsic initiation of autoimmunity. Cell 134:587–598 18724932 10.1016/j.cell.2008.06.032 PMC2626626

[23] Ablasser A, Hemmerling I, Schmid-Burgk JL, Behrendt R, Roers A, Hornung V (2014) TREX1 deficiency triggers cell-autonomous immunity in a cGAS-dependent manner. JImmunol 192:5993–5997 24813208 10.4049/jimmunol.1400737

[24] Decout A, Katz JD, Venkatraman S, Ablasser A (2021) The cGAS-STING pathway as a therapeutic target in inflammatory diseases. Nat Rev Immunol 21. 10.1038/s41577-021-00524-z 10.1038/s41577-021-00524-z PMC8029610 33833439

[25] Liu Y, Jesus AA, Marrero B, Yang D, Ramsey SE, Montealegre Sanchez GA, Tenbrock K, Wittkowski H, Jones OY, Kuehn HS, Lee CC, DiMattia MA, Cowen EW, Gonzalez B, Palmer I, DiGiovanna JJ, Biancotto A, Kim H, Tsai WL, Trier AM, Huang Y, Stone DL, Hill S, Kim HJ, St HC, Gurprasad S, Plass N, Chapelle D, Horkayne-Szakaly I, Foell D, Barysenka A, Candotti F, Holland SM, Hughes JD, Mehmet H, Issekutz AC, Raffeld M, McElwee J, Fontana JR, Minniti CP, Moir S, Kastner DL, Gadina M, Steven AC, Wingfield PT, Brooks SR, Rosenzweig SD, Fleisher TA, Deng Z, Boehm M, Paller AS, Goldbach-Mansky R (2014) Activated STING in a vascular and pulmonary syndrome. NEnglJMed 371:507–518 10.1056/NEJMoa1312625 PMC4174543 25029335

[26] Jeremiah N, Neven B, Gentili M, Callebaut I, Maschalidi S, Stolzenberg MC, Goudin N, Fremond ML, Nitschke P, Molina TJ, Blanche S, Picard C, Rice GI, Crow YJ, Manel N, Fischer A, Bader-Meunier B, Rieux-Laucat F (2014) Inherited STING-activating mutation underlies a familial inflammatory syndrome with lupus-like manifestations. JClinInvest 124:5516–5520 10.1172/JCI79100 PMC4348945 25401470

[27] König N, Fiehn C, Wolf C, Schuster M, Cura Costa E, Tüngler V, Alvarez HA, Chara O, Engel K, Goldbach-Mansky R, Günther C, Lee-Kirsch MA (2017) Familial chilblain lupus due to a gain-of-function mutation in STING. Ann Rheum Dis 76:468–472. 10.1136/annrheumdis-2016-209841 27566796 10.1136/annrheumdis-2016-209841

[28] Watkin LB, Jessen B, Wiszniewski W, Vece TJ, Jan M, Sha Y, Thamsen M, Santos-Cortez RLP, Lee K, Gambin T, Forbes LR, Law CS, Stray-Pedersen A, Cheng MH, Mace EM, Anderson MS, Liu D, Tang LF, Nicholas SK, Nahmod K, Makedonas G, Canter DL, Kwok P-Y, Hicks J, Jones KD, Penney S, Jhangiani SN, Rosenblum MD, Dell SD, Waterfield MR, Papa FR, Muzny DM, Zaitlen N, Leal SM, Gonzaga-Jauregui C, Center B-H, for Mendelian Genomics, Boerwinkle E, Eissa NT, Gibbs RA, Lupski JR, Orange JS, Shum AK (2015) COPA mutations impair ER-Golgi transport and cause hereditary autoimmune-mediated lung disease and arthritis. Nat Genet 47:654–660. 10.1038/ng.3279 25894502 10.1038/ng.3279 PMC4513663

[29] Deng Z, Chong Z, Law CS, Mukai K, Ho FO, Martinu T, Backes BJ, Eckalbar WL, Taguchi T, Shum AK (2020) A defect in COPI-mediated transport of STING causes immune dysregulation in COPA syndrome. J Exp Med 217:e20201045. 10.1084/jem.20201045 32725126 10.1084/jem.20201045 PMC7596814

[30] Lepelley A, Martin-Niclós MJ, Le Bihan M, Marsh JA, Uggenti C, Rice GI, Bondet V, Duffy D, Hertzog J, Rehwinkel J, Amselem S, Boulisfane-El Khalifi S, Brennan M, Carter E, Chatenoud L, Chhun S, Coulomb l’Hermine A, Depp M, Legendre M, Mackenzie KJ, Marey J, McDougall C, McKenzie KJ, Molina TJ, Neven B, Seabra L, Thumerelle C, Wislez M, Nathan N, Manel N, Crow YJ, Frémond M-L (2020) Mutations in COPA lead to abnormal trafficking of STING to the Golgi and interferon signaling. J Exp Med 217:e20200600. 10.1084/jem.20200600 10.1084/jem.20200600 PMC7596811 32725128

[31] Brown GJ, Cañete PF, Wang H, Medhavy A, Bones J, Roco JA, He Y, Qin Y, Cappello J, Ellyard JI, Bassett K, Shen Q, Burgio G, Zhang Y, Turnbull C, Meng X, Wu P, Cho E, Miosge LA, Andrews TD, Field MA, Tvorogov D, Lopez AF, Babon JJ, López CA, Gónzalez-Murillo Á, Garulo DC, Pascual V, Levy T, Mallack EJ, Calame DG, Lotze T, Lupski JR, Ding H, Ullah TR, Walters GD, Koina ME, Cook MC, Shen N, de Lucas CC, Corry B, Gantier MP, Athanasopoulos V, Vinuesa CG (2022) TLR7 gain-of-function genetic variation causes human lupus. Nature 605:349–356. 10.1038/s41586-022-04642-z 35477763 10.1038/s41586-022-04642-z PMC9095492

[32] Stremenova Spegarova J, Sinnappurajar P, Al Julandani D, Navickas R, Griffin H, Ahuja M, Grainger A, Livingstone K, Rice GI, Sutherland F, Hayes C, Parke S, Pang L, Roderick MR, Slatter M, Crow Y, Ramanan AV, Hambleton S (2024) A de novo TLR7 gain-of-function mutation causing severe monogenic lupus in an infant. J Clin Invest 134:e179193. 10.1172/JCI179193 38753439 10.1172/JCI179193 PMC11213501

[33] Wolf C, Lim EL, Mokhtari M, Kind B, Odainic A, Lara-Villacanas E, Koss S, Mages S, Menzel K, Engel K, Dückers G, Bernbeck B, Schneider DT, Siepermann K, Niehues T, Goetzke CC, Durek P, Minden K, Dörner T, Stittrich A, Szelinski F, Guerra GM, Massoud M, Bieringer M, de Oliveira Mann CC, Beltrán E, Kallinich T, Mashreghi M-F, Schmidt SV, Latz E, Klughammer J, Majer O, Lee-Kirsch MA (2024) UNC93B1 variants underlie TLR7-dependent autoimmunity. Sci Immunol 9:eadi9769. 10.1126/sciimmunol.adi9769 38207055 10.1126/sciimmunol.adi9769

[34] Mishra H, Schlack-Leigers C, Lim EL, Thieck O, Magg T, Raedler J, Wolf C, Klein C, Ewers H, Lee-Kirsch MA, Meierhofer D, Hauck F, Majer O (2024) Disrupted degradative sorting of TLR7 is associated with human lupus. Sci Immunol 9:eadi9575. 10.1126/sciimmunol.adi9575 38207015 10.1126/sciimmunol.adi9575

[35] Rael VE, Yano JA, Huizar JP, Slayden LC, Weiss MA, Turcotte EA, Terry JM, Zuo W, Thiffault I, Pastinen T, Farrow EG, Jenkins JL, Becker ML, Wong SC, Stevens AM, Otten C, Allenspach EJ, Bonner DE, Bernstein JA, Wheeler MT, Saxton RA, Network UD, Liu B, Majer O, Barton GM (2024) Large-scale mutational analysis identifies UNC93B1 variants that drive TLR-mediated autoimmunity in mice and humans. J Exp Med 221:e20232005. 10.1084/jem.20232005 38780621 10.1084/jem.20232005 PMC11116816

[36] Al-Azab M, Idiiatullina E, Liu Z, Lin M, Hrovat-Schaale K, Xian H, Zhu J, Yang M, Lu B, Zhao Z, Liu Y, Chang J, Li X, Guo C, Liu Y, Wu Q, Chen J, Lan C, Zeng P, Cui J, Gao X, Zhou W, Zhang Y, Zhang Y, Masters SL (2024) Genetic variants in UNC93B1 predispose to childhood-onset systemic lupus erythematosus. Nat Immunol 25:969–980. 10.1038/s41590-024-01846-5 38831104 10.1038/s41590-024-01846-5 PMC11147776

[37] David C, Arango-Franco CA, Badonyi M, Fouchet J, Rice GI, Didry-Barca B, Maisonneuve L, Seabra L, Kechiche R, Masson C, Cobat A, Abel L, Talouarn E, Béziat V, Deswarte C, Livingstone K, Paul C, Malik G, Ross A, Adam J, Walsh J, Kumar S, Bonnet D, Bodemer C, Bader-Meunier B, Marsh JA, Casanova J-L, Crow YJ, Manoury B, Frémond M-L, Bohlen J, Lepelley A (2024) Gain-of-function human UNC93B1 variants cause systemic lupus erythematosus and chilblain lupus. J Exp Med 221:e20232066. 10.1084/jem.20232066 38869500 10.1084/jem.20232066 PMC11176256

[38] Briggs TA, Rice GI, Daly S, Urquhart J, Gornall H, Bader-Meunier B, Baskar K, Baskar S, Baudouin V, Beresford MW, Black GC, Dearman RJ, de ZF, Foster ES, Frances C, Hayman AR, Hilton E, Job-Deslandre C, Kulkarni ML, Le MM, Linglart A, Lovell SC, Maurer K, Musset L, Navarro V, Picard C, Puel A, Rieux-Laucat F, Roifman CM, Scholl-Burgi S, Smith N, Szynkiewicz M, Wiedeman A, Wouters C, Zeef LA, Casanova JL, Elkon KB, Janckila A, Lebon P, Crow YJ (2011) Tartrate-resistant acid phosphatase deficiency causes a bone dysplasia with autoimmunity and a type I interferon expression signature. NatGenet 43:127–131 10.1038/ng.748 PMC3030921 21217755

[39] Shinohara ML, Lu L, Bu J, Werneck MB, Kobayashi KS, Glimcher LH, Cantor H (2006) Osteopontin expression is essential for interferon-alpha production by plasmacytoid dendritic cells. NatImmunol 7:498–506 10.1038/ni1327 PMC3725256 16604075

